# Gut Symptoms during FODMAP Restriction and Symptom Response to Food Challenges during FODMAP Reintroduction: A Real-World Evaluation in 21,462 Participants Using a Mobile Application

**DOI:** 10.3390/nu15122683

**Published:** 2023-06-09

**Authors:** Eirini Dimidi, Katerina Belogianni, Kevin Whelan, Miranda C. E. Lomer

**Affiliations:** 1 Department of Nutritional Sciences, King’s College London, London SE1 9NH, UK; 2 Department of Nutrition and Dietetics, Guy’s and St Thomas’ NHS Foundation Trust, London SE1 7EH, UK

**Keywords:** low FODMAP diet, dietary triggers, mobile apps, gut symptoms, mhealth

## Abstract

Background: There is limited evidence regarding the use of low FODMAP diet apps. This study aimed to evaluate the effectiveness of an app intended to reduce symptoms in FODMAP restriction and symptoms and tolerance of high FODMAP food challenges during FODMAP reintroduction and personalisation. Methods: Data were collected from 21,462 users of a low FODMAP diet app. Self-reported gut symptoms during FODMAP restriction, reintroduction, and personalisation and dietary triggers were identified from symptom response data for FODMAP food challenges. Results: Compared with baseline, at the end of FODMAP restriction, participants (*n* = 20,553) reported significantly less overall symptoms (11,689 (57%) versus 9105 (44%)), abdominal pain (8196 (40%) versus 6822 (33%)), bloating (11,265 (55%) versus 9146 (44%)), flatulence (10,318 (50%) 8272 (40%)), and diarrhoea (6284 (31%) versus 4961 (24%)) and significantly more constipation (5448 (27%) versus 5923 (29%)) (*p* < 0.001 for all). During FODMAP reintroduction, participants (*n* = 2053) completed 8760 food challenges; the five most frequent challenges and n/N (%) of dietary triggers identified were wheat bread 474/1146 (41%), onion 359/918 (39%), garlic 245/699 (35%), milk 274/687 (40%), and wheat pasta 222/548 (41%). The most frequently reported symptoms during food challenges were overall symptoms, abdominal pain, bloating, and flatulence. Conclusions: In a real-world setting, a low FODMAP diet app can help users improve gut symptoms and detect dietary triggers for long-term self-management.

## 1. Introduction

Many people experience gut symptoms such as diarrhoea, constipation, abdominal pain, bloating, and flatulence. These can be very common, but if they are severe and frequent enough they can lead to clinical diagnosis of disorders of gut-brain interaction that can impact quality of life [1]. A common disorder of gut-brain interaction is irritable bowel syndrome (IBS), which is chronic and relapsing, and characterised by abdominal pain, bloating, flatulence, and disordered defecation (diarrhoea and/or constipation) [1]. The prevalence of IBS is between 3.8 to 12%, depending on diagnostic criteria used and country [2,3]. It is more common in the developed world, and in women and people under 50 years old [2,4]. Its pathophysiology is not fully elucidated and involves a complex interaction between psychological (e.g., stress/distress) and biological factors such as visceral hypersensitivity, gut microbiome alterations, and brain–gut interaction [1]. Many individuals with IBS report a reduced quality of life [5,6] as well as anxiety and depression [7]. IBS management includes diet and lifestyle modifications, pharmacotherapy, or psychological treatment [8,9,10].

Adopting specific dietary approaches can significantly improve symptoms; for example, standard dietary advice (e.g., smaller frequent meals, avoiding fatty, spicy foods) is used, but where this does not improve symptoms, second line dietary advice is for dietary restriction of fermentable oligosaccharides, disaccharides, monosaccharides, and polyols (FODMAPs) [11]. Oligosaccharides include foods high in fructans (e.g., wheat, onion, garlic) and galacto-oligosaccharides (GOS, e.g., pulses, legumes, some nuts); disaccharides include foods high in lactose (i.e., milk and milk products); monosaccharides include foods high in excess fructose (e.g., mango, honey); and polyols include foods high in sorbitol (e.g., apricot, avocado) or mannitol (e.g., cauliflower, mushroom) [12].

The low FODMAP diet includes three distinctive stages: (a) FODMAP restriction, (b) FODMAP re-introduction, and (c) FODMAP personalisation, and their implementation in clinical practice has been described in detail elsewhere [12]. There have now been several randomised controlled trials of the FODMAP restriction stage of the low FODMAP diet, and systematic reviews and meta-analysis report that it significantly reduces symptoms in IBS [13,14]. Furthermore, a network analysis has shown that the low FODMAP diet ranks superior to other dietary management strategies for IBS [15]. Thus, FODMAP restriction as part of a low FODMAP diet is an effective management option and is recommended in guidelines [16,17]. However, there is almost no research on the FODMAP reintroduction stage, including what foods patients most commonly attempt to reintroduce and which foods most commonly induce symptoms.

Due to the exclusion of foods with prebiotic oligosaccharides, FODMAP restriction can negatively impact the microbiome [18], and the complex nature of the diet can impact nutrient intake (dietary fibre, calcium) and overall diet quality [19]. Following FODMAP reintroduction and FODMAP personalisation, the abundance of bifidobacteria may return to baseline levels [20]. Therefore, the importance of FODMAP reintroduction and FODMAP personalisation and of receiving guidance by a dietitian when implementing the diet has been highlighted [17,21]. 

Dietitian-led consultations can be delivered as one-to-one or group appointments [22,23,24]. However, there is a lack of dietitians who have the expertise to deliver the low FODMAP diet, so research into alternative methods of delivery is needed. Patients report that information on the low FODMAP diet provided by general practitioners and gastroenterologists is basic and they often have concerns that material from searching additional online resources is not valid nor personalised [25], and additional methods of dietary education are required. In practice, dietitians use a range of educational materials to help support patients in educating on the low FODMAP diet [12], including colour-coded sheets, online resources, and mobile applications [26]. 

The use of mobile applications (apps) in healthcare has dramatically increased over the last decade [27]. Digestive symptoms, disorders of gut-brain interaction, and IBS may be particularly amenable to app usage as those affected are often young and embracers of technology and because users may have an increased ability to better understand dietary management strategies, such as the low FODMAP diet, and to self-manage their symptoms [27]. 

Many apps on the low FODMAP diet provide detailed information regarding which foods to include and exclude during FODMAP restriction but few give comprehensive information on the reintroduction and personalisation stages of the diet [27]. Data sourced from apps can provide insight into the implementation of the low FODMAP diet in real-world settings and, in particular, on the FODMAP reintroduction and FODMAP personalisation stages that are so often missing from the research literature.

The aim of this study was to evaluate (i) the effectiveness of an app to reduce symptoms in people during FODMAP restriction; and (ii) the self-reported symptoms and tolerance of high FODMAP food challenges in people during FODMAP reintroduction and FODMAP personalisation. 

## 2. Materials and Methods

### 2.1. Participants

Individuals aged 18 years or over, who downloaded the app “FODMAP by FoodMaestro” and consented to having their data used for research purposes, were included in this retrospective service evaluation. 

The app was designed for use by individuals with IBS; however, anyone was able to download the app, so it was not possible to confirm a diagnosis of IBS. As a result, individuals with other disorders of gut-brain interaction (e.g., functional diarrhoea, functional bloating) or with minor gut symptoms not meeting diagnostic criteria (e.g., bloating, flatulence) were also included. The app allowed participants to move between stages of the low FODMAP diet based on individual needs at any given timepoint. Separate user groups were studied for each of the three stages of the low FODMAP diet. 

Eligible participants for Stage 1 (FODMAP restriction) were defined as those who completed the app-based symptom questionnaire (see outcome measures below) at baseline (at entry to FODMAP restriction) and at a subsequent time point from two weeks onwards during FODMAP restriction. 

Eligible participants for Stage 2 (FODMAP reintroduction) were defined as those who completed the app-based symptom questionnaire at baseline (at entry to FODMAP reintroduction) and at the end of Day 1 of at least one high FODMAP food challenge. Eligible participants for Stage 3 (FODMAP personalisation) were defined as those who had completed the app-based symptom questionnaire at baseline (at entry to FODMAP personalisation).

### 2.2. Ethical Considerations

All participants provided voluntary consent for their anonymised data to be used for research purposes when they downloaded and opened the app for the first time. Researchers only had access to anonymised data from participants who had consented and as this was a service evaluation of a mobile application only, ethical approval was not required.

### 2.3. Low FODMAP Diet App

The “FODMAP by FoodMaestro” app was developed as a collaboration between King’s College London (London, UK), Guy’s and St Thomas’ NHS Foundation Trust (London, UK) and FoodMaestro Ltd. (London, UK). The app enabled participants to identify suitable and unsuitable foods to follow a low FODMAP diet and record their gut symptoms during the three stages of the diet and to share this data with their healthcare professional (i.e., dietitian) if they chose. It also provided educational videos and resources for each stage of the diet.

The app provided information on suitability of foods during the low FODMAP diet. The app used a database comprising food products available in the FoodMaestro database representing the major supermarkets in the United Kingdom (e.g., ASDA, Co-op, Morrisons, Sainsbury’s, Tesco). Participants could search the database as follows:

(a) **food category**, checking the suitability of generic foods rather than specific brands for a low FODMAP diet (e.g., suitable types of cheese under dairy category);
(b) **free text,** to enable all branded food products matching free-text descriptions;
(c) **pre-determined food category groups within the app,** which generated branded food products available within that food category (e.g., suitable cheese brands under dairy category);
(d) **barcode scanner**, which used the camera function to scan the barcode of food products to identify it and display the ingredients related to that product, including whether it was suitable or not to be consumed during the low FODMAP diet.

Some foods containing FODMAPs, particularly milk and milk products and fruit and vegetables, were allowed in moderation depending on the amount of FODMAP for a pre-defined portion size of that food [28]. Thus, the suitability of food products for a low FODMAP diet was developed using algorithms and depicted using coloured face icons (red “unsuitable”; orange “in moderation”; yellow “suitable”). 

Full details of how the app supported users at each stage of the low FODMAP diet are provided in Box 1.

Box 1 **Features of the “FODMAP by FoodMaestro” app specific to each of the three stages of the low FODMAP diet.**

*App features for FODMAP restriction*

The app provided users with information to restrict high FODMAP foods for 2–8 weeks depending on their symptoms. An app-based symptom questionnaire was completed at baseline and every two weeks until the user experienced adequate symptom relief, at which point they progressed to FODMAP reintroduction, or until week eight, at which timepoint they were asked to either progress to FODMAP reintroduction (if adequate symptom relief) or stop the diet and consult their dietitian (if no adequate symptom relief).

*App features for FODMAP reintroduction*

A predefined series of high FODMAP food challenges were suggested to establish the user’s tolerance levels to that food. Prior to each food challenge, users were advised to restrict all high FODMAP foods for at least 3 days to ensure that any newly developed symptoms were due to the new food challenge. Users had the option to choose five fructan challenges (onion, garlic, wheat bread, wheat cereal, cooked wheat pasta), five GOS challenges (butter beans, canned chickpeas, peas, almond, karela), eight lactose challenges (cow, goat, or sheep milk; cow, goat, or sheep yoghurt; cottage cheese; cream cheese; ricotta; quark; ice cream; dairy milk custard), seven fructose challenges (boysenberry, broccoli, fresh fig, mango, sugar snaps, honey, agave syrup), three sorbitol challenges (avocado, blackberry, lychee), and four mannitol challenges (cauliflower, celery, sweet potato, mushrooms). Users were advised to undertake only one food challenge from each FODMAP group, except for fructans for which they were advised to attempt multiple fructan food challenges due to the wide range of degree of polymerisation of inulin type fructans and the influence of that on the rate of fermentation and gas production [12]. The app included a stepwise guide for each 3-day food challenge, including the portions to be consumed on each of the three challenge days, symptom reporting for each day, as well as tips for undertaking the challenge. Portion sizes were based on predefined tolerance thresholds for each FODMAP [28,29] for Day 1 of the challenge. Portion sizes increased in quantity to double (Day 2) and triple (Day 3) the portion size for Day 1. Users were advised to undertake at least six food challenges in total. Each challenge took up to 6 days, including 3 days for the challenge and 3 days washout if symptoms were present.

During FODMAP reintroduction, users were asked to complete the symptom questionnaire before each high FODMAP food challenge (FODMAP reintroduction baseline), and at the end of each challenge day (Day 1, Day 2, Day 3). If symptoms increased on Day 1 or Day 2 of a food challenge, users were asked to discontinue the challenge at this day, return to a low FODMAP diet and wait until their symptoms had returned to baseline levels before commencing the next food challenge.

*App features for Stage 3 (FODMAP personalization)*

FODMAP personalisation enabled users to self-manage symptoms by following as normal and varied a diet as possible, while avoiding only those FODMAP foods that triggered symptoms during the FODMAP reintroduction stage. At entry to FODMAP personalisation, users were asked to complete the symptom questionnaire one final time. Users could set up filters in the app based upon their results (i.e., tolerance levels) for food challenges undertaken during FODMAP reintroduction. This feature allowed the app to filter its database to show suitable food products to include in the diet based upon users’ individual results. For example, if a user had challenged honey (excess fructose) without triggering symptoms, all products containing excess fructose as the only high FODMAP ingredient would now be marked as “suitable” for the user. However, users could also override these filters if, for example, they had developed symptoms to a food during FODMAP reintroduction, but still wanted to include the food in their diet.

### 2.4. Outcome Measures and Data Extraction

#### 2.4.1. Gastrointestinal Symptoms

The app included a symptom questionnaire to assess self-reported severity of the following symptoms: overall symptoms, abdominal pain, bloating, flatulence, diarrhoea, and constipation using a 4 point-Likert scale rating (0 none; 1 mild; 2 moderate; 3 severe).

#### 2.4.2. FODMAP Restriction: Symptoms at Baseline and End, and the Most Common Food Searches

Data were analysed for symptoms at baseline and were compared with end of app-supported FODMAP restriction. Data on participants’ food category and food product name searches were collected. Food categories were grouped where appropriate (e.g., “fresh vegetables” and “vegetable salads” were grouped into “vegetables”). The most common free-text food searches, as well as category group searches, were also reported.

#### 2.4.3. FODMAP Reintroduction: Most Common Food Challenges and Whether Failed or Successful

The tolerance level was used to determine whether each food challenge was failed or successful via a question that appeared on the screen at the end of each day of the challenge. At the end of Days 1 and 2 participants were asked: “*Based upon your symptoms, are you happy to move to Day 2/Day 3 of the challenge?*” If participants answered ‘no’, the challenge was marked as failed (not tolerated), and participants were instructed to stop that challenge and avoid the food. At the end of Day 3 (where participants had not discontinued the food challenge on Day 1 or Day 2), participants were asked: “*During the previous three days of the challenge, do you think your symptoms have been under control?*” If participants answered ‘no’, the challenge was also marked as failed (not tolerated), and participants were instructed to avoid the food. If participants answered ‘yes’, the challenge was marked as successful (tolerated), and participants were advised to include this food in their diet after the completion of FODMAP reintroduction.

#### 2.4.4. FODMAP Personalisation: Symptom Data and FODMAP Filters

Symptom data was reported at entry to app-supported FODMAP personalisation. Foods excluded in the diet (filters on) and the most common filters turned off (overridden) in this stage were reported.

### 2.5. Statistical Analyses

Descriptive statistics were performed, and data were analysed using mean (SD) for normally distributed continuous variables, median (IQR) for non-normally distributed continuous variables, and n (%) for categorical variables. Categorical outcomes were compared between groups using the Chi-squared test (unpaired) or McNemar’s test (paired). Symptom responses were collapsed into two variables with ‘no symptoms’ representing none and mild and ‘symptoms’ representing moderate and severe. 

Cohen’s kappa coefficient was calculated to estimate the agreement (beyond chance) between food challenges, and this was performed after excluding participants reporting baseline symptoms at FODMAP reintroduction. Kappa values ≥ 0.61 to 0.80 indicated substantial agreement and kappa values ≥ 0.81 to 1.00 indicated almost perfect agreement [30].

During FODMAP reintroduction, a Cox proportional hazards regression (HR) analysis was used to assess whether the presence of symptoms (moderate/severe) during a food challenge could predict participants reporting a failed food challenge (outcome). Univariate regression analyses using Kaplan–Meier log-rank tests were performed to investigate associations between symptoms and the outcome (reporting a failed food challenge). Multivariate analyses were performed for all food challenges within each FODMAP group, with the individual symptoms as covariates, adjusting for age and sex in all models. The variable ‘overall symptoms’ was excluded from the Cox regression analyses due to multicollinearity with individual symptoms. The proportional hazard assumption was tested for all models and, when the assumption was violated, the relevant time-interaction variables were included in the final model alongside the original predictors. Data were considered significant where two-tailed *p* < 0.05. All analyses were performed with the Statistical Package for the Social Sciences (IBM SPSS Statistics for Windows, Version 27.0).

## 3. Results

Overall, 49,061 participants downloaded the app and provided consent for their data to be used for research. The number of participants eligible for analysis at any stage was 21,462, the median age was 38 (IQR 19) years, and 17,034 (80%) were female (Supplementary Information Figure S1). The remaining 27,599 participants only used the app to identify suitable and unsuitable foods and did not report symptom data for any of the stages.

### 3.1. FODMAP Restriction

**Figure 1:**
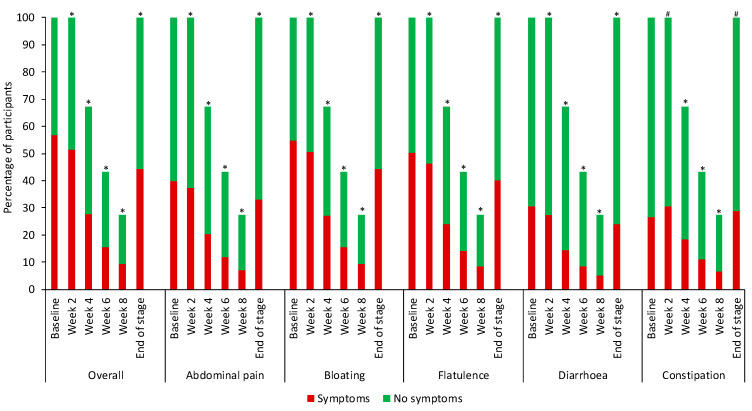
Symptom reporting for FODMAP restriction. Participants symptom reporting at baseline was overall symptoms *n* = 11,689, abdominal pain *n* = 8196, bloating *n* = 11,265, flatulence *n* = 10,318, diarrhoea *n* = 6284, constipation *n* = 5448. Using the McNemar test, * each timepoint was significantly lower compared with baseline (*p* < 0.001) # each timepoint was significantly higher compared with baseline (*p* < 0.001).

Of the 20,553 eligible participants, the median age was 38 (IQR 19) years and 16,427 (80%) were female. Participants followed FODMAP restriction for 2–8 weeks: 6733 (33%) followed the diet for 2 weeks, 4940 (24%) for 4 weeks, 3270 (16%) for 6 weeks, and 5610 (27%) for 8 weeks. Compared with baseline, by the end of app-supported FODMAP restriction (endpoint), significantly fewer participants reported overall symptoms (baseline 11,689 (57%) versus endpoint 9105 (44%) *p* < 0.001), abdominal pain (baseline 8196 (40%) versus endpoint 6822 (33%) *p* < 0.001), bloating (baseline 11,265 (55%) versus endpoint 9146 (44%) *p* < 0.001), flatulence (baseline 10,318 (50%) versus endpoint 8272 (40%) *p* < 0.001), and diarrhoea (baseline 6284 (31%) versus endpoint 4961 (24%) *p* < 0.001). However, significantly more participants reported constipation (baseline 5448 (27%) versus endpoint 5923 (29%) *p* < 0.001; Figure 1). For 15,105 (73%) participants without constipation at baseline, 2910 (14%) subsequently reported constipation at the end of FODMAP restriction. Overall, the size of the reduction was relatively small due to not all participants having symptoms at baseline. Therefore, when analysed for only those who experienced the symptom at baseline, an app-supported FODMAP restriction resulted in reductions in overall symptoms, abdominal pain, bloating, flatulence, diarrhoea, and constipation between 45% to 55% (Table 1).

**Figure 2:**
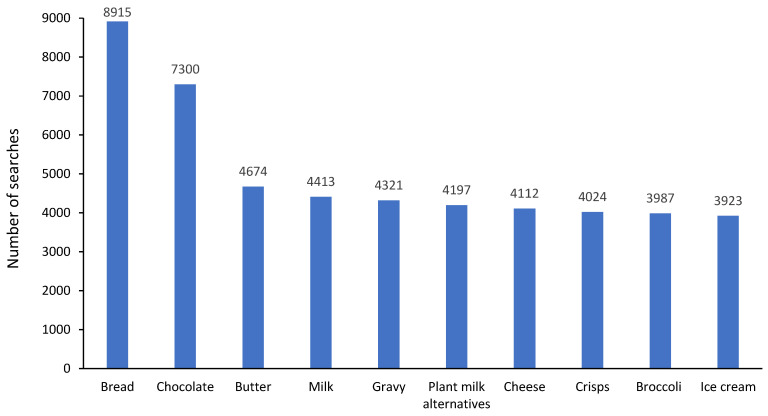
Top 10 free text foods searched for within the app regarding their suitability on a low FODMAP diet.

There were 1,118,413 free text searches from 13,496 (66%) participants with the top 10 foods searched shown in Figure 2. There were 233,453 category searches from 15,002 (73%) participants with the top five categories accounting for 63% of searches overall: 39,824 (17%) searches for bread and cereal products; 36,277 (16%) searches for cooking ingredients and condiments; 32,683 (14%) searches for fruit and vegetables; 18,782 (8%) searches for dairy; 17,730 (8%) searches for drinks. During FODMAP restriction, 17,467 (85%) participants either used free text or food category searches with a median (range) number of free text searches of 9 (0–878) per participant and category searches of 3 (0–810) per participant.

### 3.2. FODMAP Reintroduction

**Figure 3:**
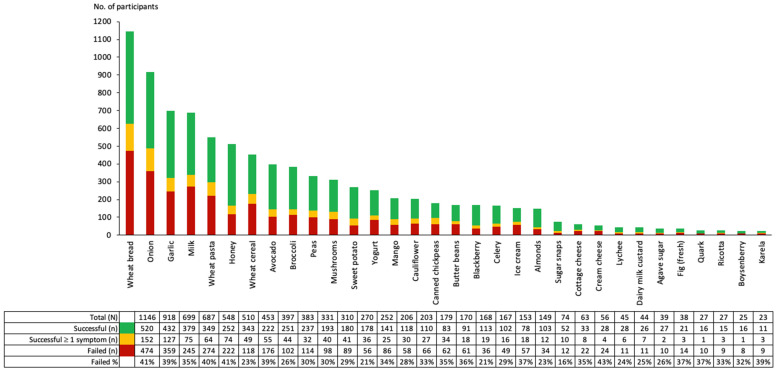
Number of participants who undertook food challenges during FODMAP reintroduction. A successful food challenge was defined as a food challenge where, on Day 3, participants chose “yes” to the question “*During the previous three days of the challenge, do you think your symptoms have been under control?*” and reported no symptoms during the challenge. A successful food challenge ≥1 symptom was defined as a food challenge where, on Day 3, participants chose “yes” to the question “*During the previous three days of the challenge, do you think your symptoms have been under control?*” and at least one symptom was reported during the three days. A failed food challenge was defined as a food challenge where, on Day 1 (or Day 2), participants chose “no” to the question “*Based upon your symptoms, are you happy to move to Day 2 (or Day 3) of the challenge?*” or, on Day 3, participants chose “no” to the question “*During the previous three days of the challenge, do you think your symptoms have been under control?*”.

In FODMAP reintroduction, 2053 participants completed 8760 food challenges in a selection of 32 different foods (Supplementary Information Table S1) with a mean number of 4.3 (SD 4.1) food challenges per participant. Participants had a median age of 39 (IQR 17) years and 1691 (82%) were female. Of the 2053 FODMAP reintroduction participants, 1435 (70%) had also completed FODMAP restriction. The most frequently reported symptoms during food challenges were overall symptoms, abdominal pain, bloating, and flatulence. Wheat bread (*n* = 1146, 13.1%), onion (*n* = 918, 10.1%), garlic (*n* = 699, 8.0%), milk (*n* = 687, 7.8%), and wheat pasta (*n* = 548, 6.3%) were the five most frequently undertaken food challenges. In addition, these foods also had the largest number of participants who failed the food challenge (n/N (%)) wheat bread (474/1146 (41%)), onion (359/918 (39%)), garlic (245/699 (35%), milk (274/687 (40%), and wheat pasta (222/548 (41%)) (Figure 3). For every single food challenge, more participants reported a successful food challenge than reported a failed food challenge (Figure 3). Not all participants who reported symptoms at the end of their food challenge reported the challenge as failed (Figure 3). For example, of the 424 (37%) participants reporting overall symptoms (moderate/severe) at the end of their wheat bread challenge, only 361 (31.5%) reported the challenge as failed (Table S1).

**Figure 4:**
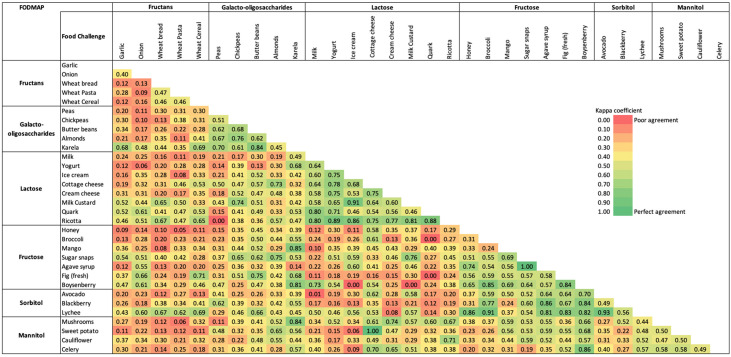
Heatmap showing kappa agreement for food challenges within and between each FODMAP group.

Overall, 3764 (43%) fructan, 852 (10%) GOS, 1309 (15%) lactose, 1275 (15%) fructose, 610 (7%) sorbitol, and 950 (11%) mannitol challenges were conducted. Among participants undertaking two food challenges from the same FODMAP group, there was almost perfect agreement in tolerance between butter beans and karela (k = 0.84, *n* = 13) (GOS challenges); yogurt and ricotta (k = 0.89, *n* = 20), ice cream and dairy milk custard (k = 0.91, *n* = 24), ice cream and ricotta (k = 0.86, *n* = 16), dairy milk custard and ricotta (k = 0.81, *n* = 13), quark and ricotta (k = 0.88, *n* = 17) (lactose challenges); sugar snaps and agave syrup (k = 1.00, *n* = 14), broccoli and boysenberry (k = 0.85, *n* = 14), fig and boysenberry (k = 0.84, *n* = 13) (fructose challenges); and avocado and lychee (k = 0.93, *n* = 29) (sorbitol challenges) (Figure 4). In addition, a substantial agreement in tolerance was found between seven GOS challenges (i.e., peas and karela, canned chickpeas and almonds, butter beans and almonds), twelve lactose challenges (i.e., milk and quark, milk and ricotta, yogurt and ice cream, yogurt and cottage cheese, yogurt and cream cheese, cottage cheese and ricotta, cream cheese and ricotta) and five fructose challenges (i.e., honey and agave syrup, mango and sugar snaps, sugar snaps and boysenberry). A slight to moderate agreement in tolerance was found between two challenges taken from the fructan group and between the mannitol group.

Among participants undertaking food challenges from different FODMAP groups, almost perfect agreement in tolerance was found between honey (fructose) and lychee (sorbitol) (k = 0.86, *n* = 19), broccoli (fructose) and lychee (sorbitol) (k = 0.91, *n* = 25), mango (fructose) and karela (GOS) (k = 0.85, *n* = 14), agave syrup (fructose) and blackberry (sorbitol) (k = 0.86, *n* = 16), agave syrup (fructose) and lychee (sorbitol) (k = 0.81, *n* = 14), fig (fructose) and lychee (sorbitol) (k = 0.83, *n* = 13), boysenberry (fructose) and karela (GOS) (k = 0.81, *n* = 11), boysenberry (fructose) and celery (mannitol) (k = 0.86, *n* = 14), boysenberry (fructose) and blackberry (sorbitol) (k = 0.84, *n* = 13), boysenberry (fructose) and lychee (sorbitol) (k = 0.82, *n* = 12), karela (GOS) and mushrooms (mannitol) (k = 0.84, *n* = 15), and cottage cheese (lactose) and sweet potato (mannitol) (k = 1.00, *n* = 16) (Figure 4).

The Cox regression analyses showed that the presence of abdominal pain, bloating, and flatulence predicted participants to report a failed food challenge for all FODMAP groups, except abdominal pain for mannitol (Table 2). The presence of diarrhoea predicted a failed food challenge for fructans (HR = 1.56, 95% CI: 1.34–1.81, *p* < 0.001), lactose (HR = 1.81, 95% CI: 1.40–2.35, *p* < 0.001), fructose (HR = 1.99, 95% CI: 1.36–2.92, *p* < 0.001), or mannitol (HR = 1.81, 95% CI: 1.24–2.64, *p* = 0.002), while the presence of constipation predicted a failed food challenge for fructans (HR = 1.20, 95% CI: 1.02–1.41, *p* = 0.028) or lactose (HR = 1.48, 95% CI: 1.12–1.94, *p* = 0.005).

### 3.3. FODMAP Personalisation

**Figure 5:**
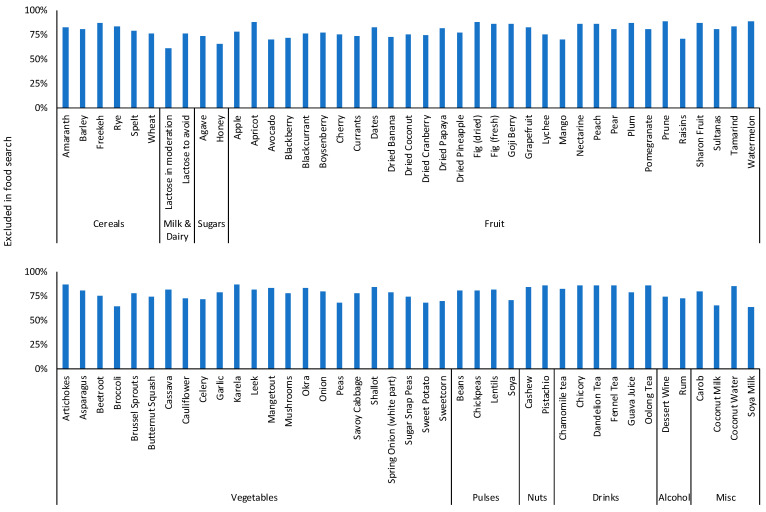
Proportion of participants in FODMAP personalisation with filters on to exclude dietary triggers in algorithm searches.

For FODMAP personalisation, data were available for 965 participants, with a median age of 42 (IQR 21) years, and 798 (83%) were female. Symptoms were only recorded by participants at entry to the FODMAP personalisation stage: overall symptoms 224 (23%); abdominal pain 171 (18%); bloating 223 (24%); flatulence 214 (22%); diarrhoea 140 (15%); constipation 144 (15%). Of the 965 FODMAP personalisation participants, 269 (28%) had also completed FODMAP restriction and FODMAP reintroduction and reported a similar level of symptoms at baseline of FODMAP restriction as the other participants who completed FODMAP restriction (overall *n* = 143 (53%), abdominal pain *n* = 107 (40%), bloating *n* = 135 (50%), flatulence *n* = 127 (47%), diarrhoea *n* = 79 (29%), constipation *n* = 60 (20%)). A further 231 (24%) had completed FODMAP restriction and skipped FODMAP reintroduction, 174 (18%) had skipped FODMAP restriction and completed FODMAP reintroduction and the remaining 291 (30%) had skipped FODMAP restriction and FODMAP reintroduction and moved straight to FODMAP personalisation. At FODMAP personalisation, the app excluded foods that were challenged and caused symptoms during FODMAP reintroduction. Therefore, all 443 participants who had completed FODMAP reintroduction had automatic filters to exclude foods in searches based on their personal dietary triggers identified during that stage (Figure 5); however, participants could also override these filters to a food if, for example, they had developed symptoms during reintroduction, but still wanted to include the food in their diet. For either of these reasons, the top 10 filters turned off (i.e., indicated that these foods were less likely to be a dietary trigger or were manually overridden by participants) were lactose in moderation for 173 (39%) participants, soya milk for 160 (36%) participants, broccoli for 159 (36%) participants, coconut milk for 155 (35%) participants, honey for 155 (35%) participants, peas for 142 (32%) participants, sweet potato for 140 (30%) participants, avocado for 134 (30%) participants, sweetcorn for 134 (30%) participants, and mango for 132 (30%) participants.

## 4. Discussion

This is the largest study to evaluate the effect of an app-supported FODMAP restriction on gut symptoms and the dietary triggers during FODMAP reintroduction. The app enabled participants to identify suitable foods and track symptoms during FODMAP restriction and provided guidance on food challenges during FODMAP reintroduction, helping participants to identify dietary triggers and individualise their diet for long-term FODMAP personalisation. 

Health-related apps are increasingly being developed to enable users to carry out a health-related intervention (e.g., the low FODMAP diet) and monitor behaviour (e.g., symptoms) with minimal direct access to healthcare systems. Healthcare professionals with clinical and research expertise in the low FODMAP diet collaborated with a digital health partner to create an app that was able to support the needs of users. The current study demonstrates that it is possible to provide information on the different stages of this complex diet and provide users with a symptom tracking system, in order to reduce symptoms during FODMAP restriction and identify their personal dietary triggers, increasing their ability to self-manage gut symptoms in the long term. 

IBS is defined using Rome IV criteria as recurrent abdominal pain with a change in defecation. However, symptoms vary widely, with diarrhoea and/or constipation being present or absent in the different subtypes, and symptoms of bloating and flatulence are often present [1]. Symptom tracking is often lacking in apps for gut disease [27]; however, this function provided an opportunity for participants to monitor their progress during the different stages of the low FODMAP diet. 

Overall symptoms, bloating, flatulence, and abdominal pain were more common at baseline than diarrhoea or constipation. In those who had symptoms, approximately half reported symptom resolution following FODMAP restriction supported by the app, particularly for overall symptoms, abdominal pain, bloating, flatulence, and diarrhoea. These findings agree with controlled clinical trials of FODMAP restriction where symptom responses between 57–68% have been reported [31,32,33,34,35,36]. Most studies assessing the clinical effectiveness of the low FODMAP diet have been dietitian-led [31,32,33,34,35,36]; however, other educational methods, including apps, are of potential use to reduce the burden on healthcare systems as dietitians are in limited supply [37,38,39], and this study shows that apps can be useful to educate people on the low FODMAP diet and improve symptoms. 

Interestingly, the current study showed that more participants reported constipation at the end of FODMAP restriction compared with baseline, and almost a fifth of participants who did not have constipation at baseline reported it at the end of FODMAP restriction. Many trials of FODMAP restriction do not include patients with constipation predominant symptoms [31,32,33,34,36] due to the potential for negative impacts of restricting high fibre foods or foods with a laxative potential (e.g., sorbitol-containing fruits) on stool frequency and consistency [12,40,41]. Thus, for both clinical consultations and apps to be effective, there needs to be information to encourage the use of suitable foods that can prevent constipation during FODMAP restriction. 

The app enabled participants to search for and identify suitable and unsuitable foods using free text and food category searches, as well as barcode scanning. Although data on the use of barcode scanning was not available, this app demonstrated that at least 85% of participants utilised the free text and food category functions. Thus, apps that incorporate a vast branded product database have the potential to increase knowledge and potentially confidence regarding which foods can be consumed during the low FODMAP diet [27,42], which is often of great concern to patients who do not have access to a dietitian [25]. 

FODMAP reintroduction is a vital part of the low FODMAP diet [12,29,43]; however, many individuals avoid challenging with high FODMAP foods, possibly due to lack of education on the 3-stage process, but also due to concerns over symptom exacerbation with dietary triggers [25,26,44]. Indeed, only a tenth of the number of participants completed FODMAP reintroduction compared with FODMAP restriction, although 77% of those who completed FODMAP reintroduction had also completed FODMAP restriction. This provides valuable data to support a need for further education on the importance of FODMAP reintroduction and further research on a less restrictive version of the diet. 

During the FODMAP challenges, participants reported symptoms, mostly abdominal pain, bloating, flatulence, and overall symptoms in response to identification of dietary triggers. Reporting symptoms in relation to self-reported tolerance of a food challenge was highly subjective. Some participants reported symptoms at the end of a food challenge but then indicated tolerance to the challenge (successful challenge). There are varying degrees to which people are able to cope with symptoms burden, e.g., one individual may be willing to have moderate constipation and bloating so long as they do not have abdominal pain, whereas another may not find it acceptable to have constipation or bloating. Some participants reported a failed challenge with only one symptom; however, it may be that other symptoms were present that were not included in the app-based symptom questionnaire. Self-reported scales are frequently used in IBS due to lack of clinical markers; while they may introduce bias and impact clinical decision making [45], in an app they provide valuable information and are less burdensome to complete [42,46]. 

There is no recommended number of foods to be challenged during FODMAP reintroduction; however, practical clinical guidance suggests one food from each FODMAP group, except for fructans where testing several foods is advised to account for the differing molecular structure [12,29]. The fructan food challenges included three different wheat products (bread, breakfast cereal, and pasta), to account for the differing food matrices which may potentially alter the bioavailability of the fructan content for each of these processed forms of wheat [47,48], as well as onion and garlic. Four of the top five dietary triggers were high fructan foods (wheat bread, wheat pasta, onion, and garlic), and lactose (milk) was the other. At a practical level, fructan foods are difficult to restrict in the diet; wheat is a major staple [49], and wheat-breads contain fructans and are the most ubiquitously consumed food in many countries [50], whilst onion and garlic add flavour and are integral to many savoury dishes [51] whether homemade or in processed food. Milk is less difficult to restrict due to the wide number of available plant-based alternative drinks (nut, oat, soya, etc.) to replace milk. In the current study, nearly half the food challenges were for fructans, which suggests that individuals wanted to know whether these foods were dietary triggers. Gluten is often blamed as the culprit of gut symptoms; however, it coexists with fructans [52], which are responsible for gut symptoms instead [53,54]. Approximately 70% of the world’s population have lactose malabsorption and tolerate up to 12 g/day lactose without generating symptoms [55]. Long-term studies of a low FODMAP diet support lactose intakes between 9.9–10.4 g/day [20,56]. Furthermore, during FODMAP personalisation, the current study showed that lactose in moderation was the most frequently filter turned off, indicating some tolerance to lactose. Similar findings for all these top dietary triggers have previously been reported following FODMAP reintroduction [56] and research on dietary triggers in individuals with IBS [57,58].

During FODMAP reintroduction, a dietary trigger was identified less than half the time, i.e., more than half of the foods challenged were tolerated by participants, indicating these foods can be included in FODMAP personalisation, which increases the number of foods that individuals can include in the long term. Many individuals avoid food challenges due to preconceived conceptions that a challenge food induces symptoms; however, a small study in 41 subjects indicated a poor correlation between perceived dietary triggers before and after following a low FODMAP diet [59]. Education on the importance of FODMAP reintroduction is crucial to ensure that individuals do not over-restrict their diet unnecessarily in the long term [12,29,56,60]. Due to increased difficulties individuals find for accessing dietitians for low FODMAP education [23,25], an app to educate people on FODMAP reintroduction is particularly appealing. 

An almost perfect level of agreement in tolerance were found when challenging lactose or fructose-containing foods, indicating that participants who tolerated one food from this group would probably tolerate other foods from the same group and vice versa. However, for fructan-containing foods, there was poor to moderate agreement in tolerance, which is in line with current practice, as dietitians recommend patients challenge with each commonly consumed high fructan food [12]; this is related to variations in fermentation of fructans with different degrees of polymerisation. A moderate agreement in tolerance was found for mannitol and GOS-containing foods and a moderate to almost perfect level of agreement was found for sorbitol-containing foods. This indicates a need for further exploration to investigate whether the tolerance to one food from each of these FODMAP groups is a good representation for all foods in the same FODMAP group. Food combinations in the same meal with differing ratios of fat, carbohydrate, and protein affect gastrointestinal motility [61], and thus the time the FODMAP may be available for absorption. The food matrix may also be an important consideration for FODMAP absorption from different foods containing that FODMAP [62]. With regard to foods containing different FODMAPs, there may be an opportunity to reduce the number of food challenges from different FODMAP groups, which would potentially reduce the duration of FODMAP reintroduction. However, due to the low number of participants undertaking some of these food challenges, the findings should be interpreted with caution. 

In a FODMAP cross-over re-challenge trial in patients with inflammatory bowel disease and functional gut symptoms, symptoms were not induced with 6 g/day GOS and 6 g/day sorbitol [63]. The sorbitol and GOS doses were more than ten times higher than that found in a typical portion of a food high in GOS or sorbitol, and the findings allude to symptom induction being due to either an additive effect when FODMAPs are consumed concurrently in the diet or a higher tolerance threshold than previously thought. Further exploration to investigate whether a low FODMAP diet needs to be so restrictive is needed [36].

Few studies have explored the induction of symptoms after challenging with whole foods containing FODMAPs, creating a real-world setting rather than an artificial one, and using specific doses of FODMAPs as previously reported in controlled trials [63,64]. Mechanistic studies show that fructans increase colonic gas and fructose increases small intestinal water [65,66]; furthermore, FODMAPs induce changes in intestinal motility [67]. However, the current study suggests there is more overlap with symptoms when real food is used to challenge, as opposed to specific doses of FODMAPs. Abdominal pain, bloating, and flatulence were significant predictors of not tolerating a food containing fructans, fructose, GOS, lactose, or sorbitol, while diarrhoea was a predictor for not tolerating a food containing fructans, fructose, or lactose and constipation was a predictor for not tolerating a food containing fructans or lactose.

### Strengths and Limitations

The app was unable to confirm a formal IBS diagnosis, which may have impacted the data and resulted in individuals with other organic causes of symptoms (e.g., coeliac disease) or with minimal/no gastrointestinal symptoms following a restrictive diet inappropriately. The conditions of use included a disclaimer indicating that the app was recommended to be used alongside dietetic education and only for individuals with IBS. Four out of five users were female. As 70% of people with IBS are female [4], this high level was not unexpected; furthermore, another study of an ehealth app in IBS reported 75% of participants as being female [68]. 

Adherence with reporting in a health app is hugely variable, and although almost 50,000 users downloaded the app, symptom data was only available for less than half of these, i.e., participants who engaged in any of the 3 stages of the low FODMAP diet. Users may download an app purely because they are interested in the information it provides; for example, in the case of this low FODMAP diet app, they may have only used the food search functions including barcode scanning. Some individuals are reluctant to report personal data, such as gastrointestinal symptoms. 

It could be argued that an app is only suitable for individuals who understand digital technology, thus limiting its use in a proportion of the population [68]. Digital users tend to be younger and IBS is most prevalent in individuals under 50 years [4]. Despite this, the top quartile of app users were between 49–89 years and older participants have also been recruited to other app studies assessing gut symptoms [68]. 

IBS is multifactorial and symptom improvement may have been due to changes in the amount of stress or anxiety rather than, or as well as, diet. Indeed, patients with IBS are at higher risk of experiencing psychological symptoms compared to healthy controls, and the low FODMAP diet has been shown to improve such symptoms [59]. However, collection of data regarding stress/anxiety was not part of the app design, as this was an app designed to provide information on a dietary treatment and assess gastrointestinal symptom responses solely.

While this is not a randomised controlled trial, a strength of this evaluation is the real-world intervention in a very large sample. Many people who experience gastrointestinal symptoms do not seek medical advice [69] and/or follow elimination diets (e.g., gluten-free) to self-manage their symptoms [70]. 

## 5. Conclusions

This app on the low FODMAP diet was designed to help users identify suitable foods, reduce symptoms, and enable food challenges and personalisation. This study provides insights into using an app to implement the different stages of the low FODMAP diet in a real-life setting to help inform clinical practice. The data shows that an app can improve symptoms during FODMAP restriction and provides sufficient detail to carry out FODMAP challenges to identify dietary triggers and safe foods for personalisation of the diet for the long term. Further evaluation of FODMAP reintroduction and personalisation will help to improve our understanding of how best to use the low FODMAP diet in IBS and other disorders of gut-brain interaction.

## Figures and Tables

**Table 1:** Participants using the app who experienced symptoms at baseline.

Symptom	Baseline n (%)	End of FODMAP Restriction n (%)	*p* Value
Overall symptoms	11,689 (100%)	6116 (52%)	<0.001
Abdominal pain	8196 (100%)	3966 (48%)	<0.001
Bloating	11,265 (100%)	6170 (55%)	<0.001
Flatulence	10,318 (100%)	5289 (51%)	<0.001
Diarrhoea	6284 (100%)	2808 (45%)	<0.001
Constipation	5448 (100%)	3013 (55%)	<0.001

**Table 2:** Cox regression models showing the presence of which symptoms predicted a failed food challenge.

Covariates *	Hazard Ratio	95% Confidence Intervals	*p* Value
Fructans (n failed = 939, N total= 2901)			
Abdominal pain	**2.36**	1.99–2.80	<0.001
Bloating	**3.12**	2.60–3.76	<0.001
Flatulence	**1.20**	1.02–1.41	0.028
Diarrhoea	**1.56**	1.34–1.81	<0.001
Constipation	**1.20**	1.02–1.41	0.028
GOS (n failed = 157, N total = 664)			
Abdominal pain	**3.35**	2.10–5.35	<0.001
Bloating	**3.05**	1.82–5.13	<0.001
Flatulence	**2.43**	1.52–3.89	<0.001
Diarrhoea	1.31	0.90–1.93	0.163
Constipation	1.28	0.84–1.93	0.252
Lactose (n failed = 325, N total = 1044)			
Abdominal pain	**3.31**	2.38–4.61	<0.001
Bloating	**2.38**	1.69–3.36	<0.001
Flatulence	**1.37**	1.05–1.78	0.021
Diarrhoea	**1.81**	1.40–2.35	<0.001
Constipation	**1.48**	1.12–1.94	0.005
Fructose (n failed = 188, N total = 1029)			
Abdominal pain	**3.07**	2.02–4.67	<0.001
Bloating	**3.99**	2.61–6.10	<0.001
Flatulence	**2.32**	1.56–3.45	<0.001
Diarrhoea	**1.99**	1.36–2.92	<0.001
Constipation	1.01	0.67–1.53	0.951
Sorbitol (n failed = 149, N total = 610)			
Abdominal pain	**2.72**	1.75–4.25	<0.001
Bloating	**3.75**	2.35–6.00	<0.001
Flatulence	**1.60**	1.01–2.51	0.044
Diarrhoea	1.39	0.90–2.15	0.141
Constipation	0.76	0.49–1.19	0.229
*Mannitol (n failed = 154, N total = 761)*			
Abdominal pain	0.76	0.49–1.17	0.205
Bloating	**3.26**	2.01–5.30	<0.001
Flatulence	**2.40**	1.52–3.78	<0.001
Diarrhoea	**1.81**	1.24–2.64	0.002
Constipation	1.14	0.74–1.75	0.553

* All models were adjusted for age and sex; reference group for each covariate was participants reporting no symptoms.

## Data Availability

Research data is available on request from the corresponding author as it is stored electronically within the University.

## Supplementary Materials

The following supporting information can be downloaded at: https://www.mdpi.com/article/10.3390/nu15122683/s1, Figure S1: Venn diagram to show participant inclusion and overlap between FODMAP restriction, FODMAP reintroduction and FODMAP personalisation; Table S1: Symptoms reported by participants at each time of point of their food challenge during FODMAP reintroduction.
