# Supplementary material for: Gut Symptoms during FODMAP Restriction and Symptom Response to Food Challenges during FODMAP Reintroduction: A Real-World Evaluation in 21,462 Participants Using a Mobile Application
Source: Nutrients. 2023 Jun 9;15(12):2683. doi: 10.3390/nu15122683 (PMC10305236; doi:10.3390/nu15122683)
Supplement: Supplementary file 1 [file nutrients-15-02683-s001.zip › nutrients-2311086-supplementary.pdf]

Figure S1. Venn diagram to show participant inclusion and overlap between FODMAP restriction, FODMAP reintroduction and FODMAP personalisation

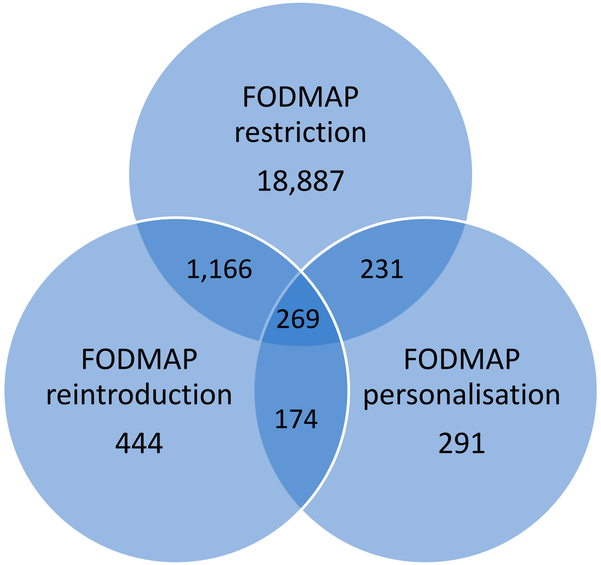

Table S1. Symptoms reported by participants at each time of point of their food challenge during FODMAP reintroduction.

Food challenge		Baseline n (%)	End of challenge (day 1, 2 or 3) n (%)		Reported symptoms at each challenge day n (%)		
		Reported Symptoms	Reported Symptoms	Failed challenge	Day 1	Day 2	Day 3
**FRUCTAN CHALLENGES**							
Garlic	*Symptoms*	*N=699*	*N=699*	*N=699*	*N=699*	*N=625*	*N=547*
	Abdominal pain	43 (6.2)	147 (21.0)	127 (18.2)	97 (13.9)	86 (13.8)	58 (10.6)
	Bloating	56 (8.0)	183 (26.2)	147 (21.0)	116 (16.6)	98 (15.7)	88 (16.1)
	Flatulence	56 (8.0)	178 (25.5)	135 (19.3)	113 (16.2)	107 (17.1)	90 (16.5)
	Diarrhoea	28 (4.0)	83 (11.9)	72 (10.3)	54 (7.7)	44 (7.0)	36 (6.6)
	Constipation	37 (5.3)	59 (8.4)	47 (6.7)	46 (6.6)	44 (7.0)	29 (5.3)
	Overall	49 (7.0)	204 (29.2)	178 (25.5)	121 (17.3)	115 (18.4)	95 (17.4)
	**Discontinued challenge** [(n (%)]			**245 (35.1)**	**74 (10.6)**	**78 (12.5)**	**93 (17.0)**
Onion	*Symptoms*	*N=918*	*N=918*	*N=918*	*N=918*	*N=790*	*N=702*
	Abdominal pain	64 (7.0)	237 (25.8)	200 (21.8)	141 (15.4)	106 (13.4)	116 (16.5)
	Bloating	76 (8.3)	279 (30.4)	219 (23.9)	152 (16.6)	138 (17.5)	150 (21.4)
	Flatulence	88 (9.6)	219 (31.7)	214 (23.3)	171 (18.6)	164 (20.8)	164 (23.4)
	Diarrhoea	41 (4.5)	122 (13.3)	99 (10.8)	69 (7.5)	61 (7.7)	65 (9.3)
	Constipation	54 (5.9)	104 (11.3)	76 (8.3)	70 (7.6)	59 (7.5)	64 (9.1)
	Overall	84 (9.2)	315 (34.3)	269 (29.3)	173 (18.8)	157 (19.9)	156 (22.2)
	**Discontinued challenge** [(n (%)]			**359 (39.1)**	**128 (139)**	**88 (11.1)**	**143 (20.4)**
Wheat bread	*Symptoms*	*N=1146*	*N=1146*	*N=1146*	*N=1146*	*N=1008*	*N=887*
	Abdominal pain	75 (6.5)	293 (25.6)	247 (21.5)	155 (13.5)	147 (14.6)	148 (16.7)
	Bloating	113 (9.9)	392 (34.2)	309 (27.0)	214 (18.7)	200 (19.8)	208 (23.4)
	Flatulence	110 (9.6)	329 (28.7)	255 (22.3)	180 (15.7)	184 (18.3)	185 (20.9)
	Diarrhoea	60 (5.2)	147 (12.8)	122 (10.6)	86 (7.5)	79 (7.8)	72 (8.1)
	Constipation	76 (6.6)	170 (14.8)	124 (10.8)	106 (9.2)	91 (9.0)	97 (10.9)
	Overall	115 (10.0)	424 (37.0)	361 (31.5)	207 (19.0)	215 (21.3)	216 (24.4)
	**Discontinued challenge** [(n (%)]			**474 (41.4)**	**138 (12.0)**	**121 (12.0)**	**215 (24.2)**

Wheat pasta	*Symptoms*	*N=548*	*N=548*	*N=548*	*N=548*	*N=468*	*N=414*
	Abdominal pain	35 (6.4)	153 (27.9)	130 (23.7)	86 (15.7)	70 (15.0)	68 (16.4)
	Bloating	51 (9.3)	189 (34.5)	151 (27.6)	111 (20.3)	87 (18.6)	97 (23.4)
	Flatulence	45 (8.2)	156 (28.5)	119 (21.7)	94 (17.2)	79 (16.9)	86 (20.8)
	Diarrhoea	26 (4.7)	78 (14.2)	64 (11.7)	46 (8.4)	37 (7.9)	35 (8.5)
	Constipation	32 (5.8)	87 (15.9)	60 (10.9)	48 (8.8)	27 (5.8)	45 (10.9)
	Overall	44 (8.0)	189 (34.5)	162 (29.6)	102 (18.6)	87 (18.6)	91 (22.0)
	**Discontinued challenge** [(n (%)]			**222 (40.5)**	**80 (14.6)**	**54 (11.5)**	**88 (21.3)**
Wheat Cereal	*Symptoms*	*N=453*	*N=453*	*N=453*	*N=453*	*N=377*	*N=345*
	Abdominal pain	34 (7.5)	109 (24.1)	96 (21.2)	71 (15.7)	36 (9.5)	48 (13.9)
	Bloating	41 (9.1)	119 (26.3)	96 (21.2)	77 (17.0)	49 (13.0)	57 (16.5)
	Flatulence	41 (9.1)	116 (25.6)	91 (20.1)	75 (16.6)	53 (13.6)	60 (17.4)
	Diarrhoea	22 (4.9)	76 (16.8)	67 (14.8)	45 (9.9)	28 (7.4)	34 (9.9)
	Constipation	30 (6.6)	58 (12.8)	42 (9.3)	49 (10.8)	28 (7.4)	31 (9.0)
	Overall	41 (9.1)	132 (29.1)	115 (25.4)	86 (19.0)	50 (13.3)	56 (16.2)
	**Discontinued challenge** [(n (%)]			**176 (38.9)**	**76 (16.8)**	**32 (8.5)**	**68 (19.7)**
**GALACTO-OLIGOSACCHARIDE CHALLENGES**							
Peas	*Symptoms*	*N=331*	*N=331*	*N=331*	*N=331*	*N=301*	*N=227*
	Abdominal pain	9 (2.7)	58 (17.5)	52 (15.7)	24 (7.3)	32 (10.6)	27 (9.7)
	Bloating	17 (5.1)	67 (20.2)	49 (14.2)	28 (8.5)	37 (12.3)	44 (15.9)
	Flatulence	10 (3.0)	74 (22.4)	49 (14.2)	33 (10.0)	38 (12.6)	55 (19.9)
	Diarrhoea	13 (3.9)	26 (7.9)	19 (5.7)	17 (5.1)	12 (4.0)	17 (6.1)
	Constipation	9 (2.7)	20 (6.0)	13 (3.9)	17 (5.1)	14 (4.7)	14 (5.1)
	Overall	14 (4.2)	75 (22.7)	63 (19.0)	35 (10.6)	36 (12.0)	42 (15.2)
	**Discontinued challenge** [(n (%)]			**98 (29.6)**	**30 (9.1)**	**24 (8.0)**	**44 (19.4)**
Canned chickpeas	*Symptoms*	*N=179*	*N=179*	*N=179*	*N=179*	*N=162*	*N=139*
	Abdominal pain	15 (8.4)	44 (24.6)	36 (20.1)	23 (12.8)	22 (13.6)	20 (14.4)
	Bloating	20 (11.2)	62 (34.6)	47 (26.3)	33 (18.4)	32 (19.8)	28 (20.1)

	Flatulence	17 (9.5)	66 (36.9)	43 (24.0)	34 (19.0)	38 (23.5)	34 (24.5)
	Diarrhoea	6 (3.4)	21 (11.7)	19 (10.6)	9 (5.0)	9 (5.6)	11 (7.9)
	Constipation	12 (6.7)	20 (11.2)	14 (7.8)	14 (7.8)	14 (8.6)	11 (7.9)
	Overall	19 (10.6)	61 (34.1)	48 (26.8)	28 (15.6)	32 (19.8)	27 (19.4)
	**Discontinued challenge** [(n (%)]			**62 (34.6)**	**17 (9.5)**	**23 (14.2)**	**22 (15.8)**
Butter beans	*Symptoms*	*N=170*	*N=170*	*N=170*	*N=170*	*N=142*	*N=129*
	Abdominal pain	12 (7.1)	35 (20.6)	34 (20.0)	22 (12.9)	15 (10.6)	10 (7.8)
	Bloating	18 (10.6)	44 (25.9)	36 (21.2)	28 (16.5)	16 (11.3)	18 (14.0)
	Flatulence	12 (7.1)	45 (26.5)	37 (21.8)	32 (18.8)	20 (14.1)	20 (15.5)
	Diarrhoea	6 (3.5)	19 (11.2)	17 (10.0)	10 (5.9)	7 (4.9)	5 (3.9)
	Constipation	8 (4.7)	18 (10.6)	12 (7.1)	13 (7.6)	8 (5.6)	9 (7.0)
	Overall	16 (9.4)	54 (31.8)	49 (28.8)	31 (18.2)	18 (12.7)	21 (16.3)
	**Discontinued challenge** [(n (%)]			**61 (35.9)**	**28 (16.5)**	**13 (9.2)**	**20 (15.5)**
Almonds	*Symptoms*	*N=149*	*N=149*	*N=149*	*N=149*	*N=132*	*N=129*
	Abdominal pain	6 (4.0)	18 (12.1)	16 (10.7)	17 (11.4)	7 (5.3)	7 (5.4)
	Bloating	10 (6.7)	21 (14.1)	16 (10.7)	18 (12.1)	6 (4.5)	9 (7.0)
	Flatulence	12 (8.1)	18 (12.1)	12 (8.1)	14 (9.4)	6 (4.5)	9 (7.0)
	Diarrhea	3 (2.0)	8 (5.4)	8 (5.4)	6 (4.0)	1 (0.8)	3 (2.3)
	Constipation	3 (2.0)	13 (8.7)	9 (6.0)	13 (8.7)	7 (5.3)	7 (5.4)
	Overall	7 (4.7)	24 (16.1)	20 (13.4)	21 (14.1)	8 (6.1)	11 (8.5)
	**Discontinued challenge** [(n (%)]			**34 (22.8)**	**17 (11.4)**	**3 (2.3)**	**14 (10.9)**
Karela	*Symptoms*	*N=23*	*N=23*	*N=23*	*N=23*	*N=16*	*N=15*
	Abdominal pain	3 (13.0)	9 (39.1)	8 (34.8)	8 (34.8)	2 (12.5)	2 (13.3)
	Bloating	3 (13.0)	9 (39.1)	9 (39.1)	9 (39.1)	2 (12.5)	1 (6.7)
	Flatulence	5 (21.7)	9 (39.1)	7 (30.4)	8 (34.8)	3 (18.8)	3 (20.0)
	Diarrhoea	3 (13.0)	8 (34.8)	7 (30.4)	7 (30.4)	2 (12.5)	2 (13.3)
	Constipation	2 (8.7)	6 (26.1)	4 (17.4)	4 (17.4)	2 (12.5)	2 (13.3)
	Overall	3 (13.0)	8 (34.8)	8 (34.8)	8 (34.8)	2 (12.5)	1 (6.7)

	**Discontinued challenge** [(n (%)]			**9 (39.1)**	**7 (30.4)**	**1 (6.3)**	**1 (6.7)**
**LACTOSE CHALLENGES**							
Milk (cow, goat or sheep)	*Symptoms*	*N=687*	*N=687*	*N=687*	*N=687*	*N=596*	*N=517*
	Abdominal pain	42 (6.1)	173 (25.2)	157 (22.9)	105 (15.3)	91 (15.3)	69 (13.3)
	Bloating	47 (6.8)	175 (25.5)	148 (21.5)	107 (15.6)	94 (16.8)	73 (14.1)
	Flatulence	54 (7.9)	196 (28.5)	164 (23.9)	115 (16.7)	113 (19.0)	91 (17.6)
	Diarrhoea	28 (4.1)	109 (15.9)	97 (14.1)	67 (9.8)	41 (6.9)	39 (7.5)
	Constipation	37 (5.4)	72 (10.5)	55 (8.0)	44 (6.4)	33 (5.5)	35 (6.8)
	Overall	48 (7.0)	231 (33.6)	204 (29.7)	121 (17.6)	121 (20.3)	96 (18.6)
	**Discontinued challenge** [(n (%)]			**274 (39.9)**	**91 (13.2)**	**79 (13.3)**	**104 (20.1)**
Yogurt (cow, goat or sheep)	*Symptoms*	*N=252*	*N=252*	*N=252*	*N=252*	*N=220*	*N=192*
	Abdominal pain	16 (6.3)	56 (22.2)	48 (19.0)	43 (17.1)	27 (12.3)	20 (10.4)
	Bloating	21 (8.3)	68 (27.0)	57 (22.6)	42 (16.7)	36 (16.4)	30 (15.6)
	Flatulence	20 (7.9)	55 (21.8)	39 (15.5)	33 (13.1)	30 (13.6)	30 (15.6)
	Diarrhoea	11 (4.4)	35 (13.9)	32 (12.7)	29 (11.5)	12 (5.5)	9 (4.7)
	Constipation	22 (8.7)	30 (11.9)	26 (10.3)	27 (10.7)	11 (5.0)	15 (7.8)
	Overall	28 (11.1)	70 (27.8)	59 (23.4)	48 (19.0)	39 (17.7)	28 (14.6)
	**Discontinued challenge** [(n (%)]			**86 (34.1)**	**32 (12.7)**	**28 (12.7)**	**26 (13.5)**
Cottage cheese	*Symptoms*	*N=63*	*N=63*	*N=63*	*N=63*	*N=45*	*N=43*
	Abdominal pain	8 (12.7)	22 (34.9)	17 (27.0)	16 (25.4)	3 (6.7)	7 (16.3)
	Bloating	6 (9.5)	19 (30.2)	16 (25.4)	15 (23.8)	3 (6.7)	5 (11.6)
	Flatulence	6 (9.5)	16 (25.4)	12 (19.0)	12 (19.0)	5 (11.1)	6 (14.0)
	Diarrhoea	3 (4.8)	16 (25.4)	14 (22.2)	15 (23.8)	3 (6.7)	3 (7.0)
	Constipation	8 (12.7)	11 (17.5)	9 (14.3)	12 (19.0)	2 (4.4)	2 (4.7)
	Overall	9 (14.3)	21 (33.3)	17 (27.0)	16 (25.4)	4 (8.9)	6 (14.0)
	**Discontinued challenge** [(n (%)]			**22 (34.9)**	**18 (28.6)**	**2 (4.4)**	**2 (4.7)**
Cream cheese	*Symptoms*	*N=56*	*N=56*	*N=56*	*N=56*	*N=39*	*N=37*
	Abdominal pain	7 (7.1)	19 (33.9)	17 (30.4)	14 (25.0)	3 (7.7)	6 (16.2)

	Bloating	5 (8.9)	14 (25.0)	12 (21.4)	12 (21.4)	2 (5.1)	3 (8.1)
	Flatulence	5 (8.9)	14 (25.0)	11 (19.6)	10 (17.9)	5 (12.8)	6 (16.2)
	Diarrhoea	3 (5.4)	12 (21.4)	10 (17.9)	9 (16.1)	4 (10.3)	4 (10.8)
	Constipation	6 (10.7)	11 (19.6)	10 (17.9)	11 (19.6)	0 (0.0)	1 (2.7)
	Overall	7 (12.5)	23 (41.1)	21 (37.5)	16 (28.6)	3 (7.7)	6 (16.2)
	**Discontinued challenge** [(n (%)]			**24 (42.9)**	**17 (30.4)**	**2 (5.1)**	**5 (13.5)**
Quark	*Symptoms*	*N=27*	*N=27*	*N=27*	*N=27*	*N=19*	*N=18*
	Abdominal pain	3 (11.1)	7 (25.9)	7 (25.9)	8 (29.6)	1 (5.3)	1 (5.6)
	Bloating	3 (11.1)	6 (22.2)	6 (22.2)	6 (22.2)	1 (5.3)	1 (5.6)
	Flatulence	3 (11.1)	8 (29.6)	7 (25.9)	8 (29.6)	3 (15.8)	2 (11.1)
	Diarrhoea	2 (7.4)	6 (22.2)	6 (22.2)	6 (22.2)	0 (0.0)	0 (0.0)
	Constipation	3 (11.1)	5 (18.5)	4 (14.8)	5 (18.5)	2 (10.5)	1 (5.6)
	Overall	3 (11.1)	8 (29.6)	8 (29.6)	8 (29.6)	1 (5.3)	1 (5.6)
	**Discontinued challenge** [(n (%)]			**10 (37.0)**	**8 (29.6)**	**1 (5.3)**	**1 (5.6)**
Ricotta	*Symptoms*	*N=27*	*N=27*	*N=27*	*N=27*	*N=20*	*N=18*
	Abdominal pain	1 (3.7)	5 (18.5)	5 (18.5)	6 (22.2)	1 (5.0)	0 (0.0)
	Bloating	5 (18.5)	8 (29.6)	7 (25.9)	8 (29.6)	2 (10.0)	1 (5.6)
	Flatulence	3 (11.1)	8 (29.6)	6 (22.2)	7 (25.9)	2 (10.0)	2 (11.1)
	Diarrhoea	1 (3.7)	7 (25.9)	6 (22.2)	7 (25.9)	2 (10.0)	1 (5.6)
	Constipation	3 (11.1)	5 (18.5)	5 (18.5)	5 (18.5)	1 (5.0)	0 (0.0)
	Overall	3 (11.1)	8 (29.6)	8 (29.6)	9 (33.3)	1 (5.0)	0 (0.0)
	**Discontinued challenge** [(n (%)]			**9 (33.3)**	**7 (25.9)**	**2 (10.0)**	**0 (0.0)**
Ice cream	*Symptoms*	*N=153*	*N=153*	*N=153*	*N=153*	*N=132*	*N=118*
	Abdominal pain	10 (6.5)	46 (30.1)	44 (28.8)	24 (15.7)	22 (16.7)	20 (16.9)
	Bloating	12 (7.8)	44 (28.8)	39 (25.5)	30 (19.6)	21 (15.9)	17 (14.4)
	Flatulence	14 (9.2)	49 (32.0)	39 (25.5)	27 (17.6)	24 (18.2)	25 (21.2)
	Diarrhoea	11 (7.2)	29 (19.0)	23 (15.0)	16 (10.5)	10 (7.6)	16 (13.6)
	Constipation	7 (4.6)	20 (13.1)	17 (11.1)	11 (7.2)	12 (9.1)	8 (6.8)

	Overall	12 (7.8)	53 (34.6)	48 (31.4)	29 (19.0)	30 (22.7)	23 (19.5)
	**Discontinued challenge** [(n (%)]			**57 (37.3)**	**21 (13.7)**	**14 (10.6)**	**22 (18.6)**
Dairy milk custard	*Symptoms*	*N=44*	*N=44*	*N=44*	*N=44*	*N=36*	*N=36*
	Abdominal pain	2 (4.5)	9 (20.5)	7 (15.9)	7 (15.9)	1 (2.8)	4 (11.1)
	Bloating	3 (6.8)	8 (19.2)	5 (11.4)	7 (15.9)	3 (8.3)	4 (11.1)
	Flatulence	3 (6.8)	9 (20.5)	5 (11.4)	7 (15.9)	2 (5.6)	5 (13.9)
	Diarrhoea	2 (4.5)	10 (22.7)	7 (15.9)	7 (15.9)	4 (11.1)	6 (16.7)
	Constipation	3 (6.8)	5 (11.4)	4 (9.1)	5 (11.4)	1 (2.8)	1 (2.8)
	Overall	3 (6.8)	9 (20.5)	7 (15.9)	7 (15.9)	3 (8.3)	4 (11.1)
	**Discontinued challenge** [(n (%)]			**11 (25.0)**	**8 (18.2)**	**0 (0.0)**	**3 (8.3)**
**FRUCTOSE CHALLENGES**							
Honey	*Symptoms*	*N=510*	*N=510*	*N=510*	*N=510*	*N=471*	*N=445*
	Abdominal pain	29 (5.7)	66 (12.9)	54 (10.6)	36 (7.1)	31 (6.6)	36 (8.1)
	Bloating	34 (6.7)	75 (14.7)	56 (11.0)	51 (10.0)	35 (7.4)	38 (8.5)
	Flatulence	29 (5.7)	70 (13.7)	50 (9.8)	44 (8.6)	36 (7.6)	40 (9.0)
	Diarrhoea	16 (3.1)	32 (6.3)	26 (5.1)	19 (3.7)	19 (4.0)	18 (4.0)
	Constipation	23 (4.5)	38 (7.5)	22 (4.3)	27 (5.3)	22 (4.7)	23 (5.2)
	Overall	38 (7.5)	101 (19.8)	77 (15.1)	49 (9.6)	43 (9.1)	57 (12.8)
	**Discontinued challenge** [(n (%)]			**118 (23.1)**	**39 (7.6)**	**26 (5.5)**	**53 (11.9)**
Broccoli	*Symptoms*	*N=383*	*N=383*	*N=383*	*N=383*	*N=345*	*N=310*
	Abdominal pain	22 (5.7)	69 (18.0)	58 (15.1)	29 (8.4)	31 (10.0)	69 (18.0)
	Bloating	31 (8.1)	80 (20.9)	69 (18.0)	44 (12.8)	34 (11.0)	80 (20.9)
	Flatulence	32 (8.4)	78 (20.4)	62 (16.2)	43 (12.5)	39 (12.6)	78 (20.4)
	Diarrhoea	18 (4.7)	33 (8.6)	30 (7.8)	18 (5.2)	14 (4.5)	33 (8.6)
	Constipation	18 (4.7)	23 (6.0)	16 (4.2)	18 (5.2)	14 (4.5)	23 (6.0)
	Overall	28 (7.3)	82 (21.4)	75 (19.6)	39 (11.3)	33 (10.6)	82 (21.4)
	**Discontinued challenge** [(n (%)]			**114 (29.8)**	**38 (9.9)**	**35 (10.1)**	**41 (13.2)**
Mango	*Symptoms*	*N=206*	*N=206*	*N=206*	*N=206*	*N=193*	*N=174*
	Abdominal pain	8 (3.9)	41 (19.9)	36 (17.5)	20 (9.7)	25 (13.0)	21 (12.1)

	Bloating	14 (6.8)	44 (21.4)	33 (16.0)	25 (12.1)	29 (15.0)	26 (14.9)
	Flatulence	8 (3.9)	50 (24.3)	35 (17.0)	20 (9.7)	31 (16.1)	29 (16.7)
	Diarrhoea	3 (1.5)	17 (8.3)	14 (6.8)	6 (2.9)	11 (5.7)	18 (4.0)
	Constipation	12 (5.8)	23 (11.2)	16 (7.8)	16 (7.8)	14 (7.3)	15 (8.6)
	Overall	11 (5.3)	60 (29.1)	45 (21.8)	24 (11.7)	30 (15.5)	35 (20.1)
	**Ended challenge** [(n (%)]			**58 (28.2)**	**13 (6.3)**	**19 (9.8)**	**26 (14.9)**
Sugar snaps	*Symptoms*	*N=74*	*N=74*	*N=74*	*N=74*	*N=69*	*N=66*
	Abdominal pain	3 (4.1)	7 (9.5)	5 (6.8)	4 (5.4)	3 (4.3)	3 (4.5)
	Bloating	5 (6.8)	10 (13.5)	7 (9.5)	6 (8.1)	4 (5.8)	6 (9.1)
	Flatulence	4 (5.4)	10 (13.5)	5 (6.8)	6 (8.1)	4 (5.8)	6 (9.1)
	Diarrhoea	4 (5.4)	5 (6.8)	4 (5.4)	4 (5.4)	2 (2.9)	2 (3.0)
	Constipation	2 (2.7)	4 (5.4)	1 (1.4)	2 (2.7)	2 (2.9)	3 (4.5)
	Overall	4 (5.4)	10 (13.5)	8 (10.8)	4 (5.4)	4 (5.8)	5 (7.6)
	**Discontinued challenge** [(n (%)]			**12 (16.2)**	**5 (6.8)**	**3 (4.3)**	**4 (6.1)**
Agave syrup	*Symptoms*	*N=39*	*N=39*	*N=39*	*N=39*	*N=33*	*N=31*
	Abdominal pain	3 (7.7)	4 (10.3)	4 (10.3)	4 (10.3)	0 (0.0)	1 (3.2)
	Bloating	3 (7.7)	5 (12.8)	4 (10.3)	5 (12.8)	1 (3.0)	2 (6.5)
	Flatulence	4 (10.3)	6 (15.4)	4 (10.3)	7 (17.9)	5 (15.2)	3 (9.7)
	Diarrhoea	3 (7.7)	3 (7.7)	3 (7.7)	3 (7.7)	0 (0.0)	0 (0.0)
	Constipation	4 (10.3)	3 (7.7)	3 (7.7)	4 (10.3)	2 (6.1)	1 (3.2)
	Overall	3 (7.7)	4 (10.3)	4 (10.3)	5 (12.8)	0 (0.0)	1 (3.2)
	**Discontinued challenge** [(n (%)]			**10 (25.6)**	**6 (15.4)**	**2 (6.1)**	**2 (6.5)**
Fig (fresh)	*Symptoms*	*N=38*	*N=38*	*N=38*	*N=38*	*N=32*	*N=29*
	Abdominal pain	1 (2.6)	7 (18.4)	7 (18.4)	6 (15.8)	2 (6.3)	2 (6.9)
	Bloating	3 (7.9)	11 (28.9)	9 (23.7)	7 (18.4)	5 (15.6)	5 (17.2)
	Flatulence	5 (13.2)	9 (23.7)	8 (21.1)	8 (21.1)	4 (12.5)	3 (10.3)
	Diarrhoea	0 (0.0)	4 (10.5)	4 (10.5)	3 (7.9)	2 (6.3)	2 (6.9)
	Constipation	2 (5.3)	8 (21.1)	6 (15.8)	6 (15.8)	2 (6.3)	5 (17.2)

	Overall	2 (5.3)	10 (26.3)	10 (26.3)	7 (18.4)	4 (12.5)	4 (13.8)
	**Discontinued challenge** [(n (%)]			**14 (36.8)**	**6 (15.8)**	**3 (9.4)**	**5 (17.2)**
Boysenberry	*Symptoms*	*N=25*	*N=25*	*N=25*	*N=25*	*N=18*	*N=18*
	Abdominal pain	0 (0.0)	2 (8.0)	2 (8.0)	2 (8.0)	0 (0.0)	0 (0.0)
	Bloating	0 (0.0)	2 (8.0)	2 (8.0)	3 (12.0)	0 (0.0)	0 (0.0)
	Flatulence	2 (8.0)	3 (12.0)	2 (8.0)	3 (12.0)	0 (0.0)	1 (5.6)
	Diarrhoea	1 (4.0)	2 (8.0)	2 (8.0)	2 (8.0)	0 (0.0)	0 (0.0)
	Constipation	2 (8.0)	1 (4.0)	1 (4.0)	3 (12.0)	0 (0.0)	0 (0.0)
	Overall	1 (4.0)	3 (12.0)	3 (12.0)	3 (12.0)	0 (0.0)	0 (0.0)
	**Discontinued challenge** [(n (%)]			**8 (32.0)**	**7 (28)**	**0 (0.0)**	**1 (5.6)**
**SORBITOL (POLYOL) CHALLENGES**							
Avocado	*Symptoms*	*N=397*	*N=397*	*N=397*	*N=397*	*N=362*	*N=337*
	Abdominal pain	21 (5.3)	62 (15.6)	52 (13.1)	37 (9.3)	27 (705)	31 (9.2)
	Bloating	34 (8.6)	78 (19.6)	59 (14.9)	50 (12.6)	37 (10.2)	42 (12.5)
	Flatulence	24 (6.0)	75 (18.9)	53 (13.4)	42 (10.6)	33 (9.1)	43 (12.8)
	Diarrhoea	9 (2.3)	20 (5.0)	14 (3.5)	17 (4.3)	11 (3.0)	8 (2.4)
	Constipation	26 (6.5)	35 (8.8)	20 (5.0)	25 (6.3)	21 (5.8)	22 (6.5)
	Overall	26 (6.5)	79 (19.9)	66 (16.6)	41 (10.3)	33 (9.1)	39 (11.6)
	**Discontinued challenge** [(n (%)]			**102 (25.7)**	**35 (8.8)**	**25 (6.9)**	**42 (12.5)**
Blackberry	*Symptoms*	*N=168*	*N=168*	*N=168*	*N=168*	*N=158*	*N=151*
	Abdominal pain	7 (4.2)	24 (14.3)	19 (11.3)	13 (7.7)	14 (8.9)	16 (10.6)
	Bloating	13 (7.7)	36 (21.4)	26 (15.5)	22 (13.1)	19 (12.0)	23 (15.2)
	Flatulence	7 (4.2)	28 (16.7)	19 (11.3)	17 (10.1)	15 (9.5)	16 (10.6)
	Diarrhoea	7 (4.2)	17 (10.1)	15 (8.9)	12 (7.1)	8 (5.1)	9 (6.0)
	Constipation	11 (6.5)	15 (8.9)	10 (6.0)	13 (7.7)	9 (5.7)	10 (6.6)
	Overall	14 (8.3)	35 (20.8)	27 (16.1)	18 (10.7)	13 (8.2)	22 (14.6)
	**Discontinued challenge** [(n (%)]			**36 (21.4)**	**10 (6.0)**	**7 (4.4)**	**19 (12.6)**
Lychee	*Symptoms*	*N=45*	*N=45*	*N=45*	*N=45*	*N=36*	*N=35*

	Abdominal pain	6 (13.3)	10 (22.2)	7 (15.6)	8 (17.8)	2 (5.6)	4 (11.4)
	Bloating	9 (20.0)	12 (26.7)	7 (15.6)	9 (20.0)	4 (11.1)	6 (17.1)
	Flatulence	9 (20.0)	12 (26.7)	7 (15.6)	9 (20.0)	5 (13.9)	6 (17.1)
	Diarrhoea	4 (8.9)	7 (15.6)	5 (11.1)	5 (11.1)	2 (5.6)	3 (8.6)
	Constipation	6 (13.3)	7 (15.6)	4 (8.9)	4 (8.9)	2 (5.6)	4 (11.4)
	Overall	8 (17.8)	41 (31.1)	8 (17.8)	10 (22.2)	4 (11.1)	7 (20.0)
	**Discontinued challenge** [(n (%)]			**11 (24.4)**	**9 (20.0)**	**1 (2.8)**	**1 (2.9)**
**MANNITOL (POLYOL) CHALLENGES**							
Mushrooms	*Symptoms*	*N=310*	*N=310*	*N=310*	*N=310*	*N=276*	*N=260*
	Abdominal pain	16 (5.2)	61 (19.7)	52 (16.8)	37 (11.9)	30 (10.9)	26 (10.0)
	Bloating	28 (9.0)	68 (21.9)	53 (17.1)	38 (12.3)	33 (12.0)	36 (13.8)
	Flatulence	20 (6.5)	66 (21.3)	43 (13.9)	39 (12.6)	36 (13.0)	38 (14.6)
	Diarrhoea	19 (6.1)	32 (10.3)	23 (7.4)	17 (5.5)	14 (5.1)	17 (6.5)
	Constipation	16 (5.2)	37 (11.9)	23 (7.4)	23 (7.4)	21 (7.6)	25 (9.6)
	Overall	19 (6.1)	72 (23.2)	59 (19.0)	33 (10.6)	31 (11.2)	37 (14.2)
	**Discontinued challenge** [(n (%)]			**89 (28.7)**	**34 (11.0)**	**16 (5.8)**	**39 (15.0)**
Sweet potato	*Symptoms*	*N=270*	*N=270*	*N=270*	*N=270*	*N=248*	*N=235*
	Abdominal pain	12 (4.4)	38 (14.1)	31 (11.5)	22 (8.1)	12 (4.8)	17 (7.2)
	Bloating	18 (6.7)	48 (17.8)	31 (11.5)	30 (11.1)	21 (8.5)	24 (10.2)
	Flatulence	13 (4.8)	47 (17.4)	31 (11.5)	33 (12.2)	23 (9.3)	27 (11.5)
	Diarrhoea	8 (3.0)	25 (9.3)	20 (7.4)	12 (4.4)	10 (4.0)	12 (5.1)
	Constipation	6 (2.2)	22 (8.1)	15 (5.6)	11 (4.1)	10 (1.0)	13 (5.5)
	Overall	14 (5.2)	51 (18.9)	38 (14.1)	31 (11.5)	21 (8.5)	25 (10.6)
	**Discontinued challenge** [(n (%)]			**56 (20.7)**	**22 (8.1)**	**13 (5.2)**	**21 (8.9)**
Cauliflower	*Symptoms*	*N=203*	*N=203*	*N=203*	*N=203*	*N=173*	*N=157*
	Abdominal pain	17 (8.4)	49 (24.1)	39 (19.2)	39 (19.2)	21 (12.1)	18 (11.5)
	Bloating	26 (12.8)	58 (28.6)	45 (22.2)	42 (20.7)	26 (15.0)	27 (17.2)
	Flatulence	22 (10.8)	54 (26.6)	42 (20.7)	39 (19.2)	32 (18.5)	27 (17.2)

	Diarrhoea	9 (4.4)	22 (10.8)	18 (8.9)	15 (7.4)	9 (5.2)	10 (6.4)
	Constipation	14 (6.9)	27 (13.3)	16 (7.9)	20 (9.9)	15 (8.7)	16 (10.2)
	Overall	22 (10.8)	62 (30.5)	50 (24.6)	42 (20.7)	27 (15.6)	27 (17.2)
	**Discontinued challenge** [(n (%)]			**66 (32.5)**	**30 (14.8)**	**16 (9.2)**	**20 (12.7)**
Celery	*Symptoms*	*N=167*	*N=167*	*N=167*	*N=167*	*N=150*	*N=137*
	Abdominal pain	8 (4.8)	26 (15.6)	24 (14.4)	15 (9.0)	14 (9.3)	11 (8.0)
	Bloating	10 (6.0)	31 (18.6)	22 (13.2)	18 (10.8)	14 (9.3)	16 (11.7)
	Flatulence	7 (4.2)	30 (18.0)	24 (13.4)	13 (7.8)	18 (12.0)	16 (11.7)
	Diarrhoea	1 (0.6)	14 (8.4)	11 (6.6)	6 (3.6)	6 (4.0)	7 (5.1)
	Constipation	9 (5.4)	11 (6.6)	7 (4.2)	9 (5.4)	11 (7.3)	4 (2.9)
	Overall	11 (6.6)	35 (21.0)	30 (18.0)	18 (10.8)	16 (10.7)	18 (13.1)
	**Discontinued challenge** [(n (%)]			**49 (29.3)**	**17 (10.2)**	**13 (8.7)**	**19 (13.9)**
